# Pre-ascent hypoxic preparedness for rapid-ascent mountain sports: translating military evidence into civilian acclimatization research

**DOI:** 10.3389/fphys.2026.1935543

**Published:** 2026-09-03

**Authors:** Wei-Long Zhang, Kai-Yun Dou, Yang Zhang

**Affiliations:** 1 College of Physical Education, Hunan Normal University, Changsha, Hunan, China; 2 Hebei Mental Health Center, Baoding, Hebei, China; 3 Independent Person, Windermere, FL, United States

**Keywords:** acute mountain sickness, high-altitude travel, hypoxic pre-acclimatization, intermittent hypoxia, normobaric hypoxia

## 1 Introduction

Mountain sports increasingly combine rapid access with early exertion. Trekking, skiing, mountaineering, trail running, and recreational travel may begin soon after ascent by road, rail, cableway, or air. Tibet illustrates the scale of contemporary high-altitude tourism in China. In 2025, it received more than 70.73 million domestic and international visitors, many of whom entered high-altitude towns or scenic routes within short travel itineraries (Xinhua News Agency, 2026). The resulting mismatch is physiological: access has accelerated, but acclimatization still unfolds over days.

Acute mountain sickness (AMS) remains the most common clinical problem during early exposure to altitude. Symptoms can disturb sleep and appetite and can limit mobility during a trip. They may also complicate participation in mountain activities when exertion and limited medical access are involved. High-altitude cerebral edema and high-altitude pulmonary edema are less common but potentially life-threatening (Bärtsch and Swenson, 2013). Established prevention therefore emphasizes gradual ascent, staged acclimatization, conservative exertion early after arrival, and acetazolamide for selected risk profiles. Descent or oxygen remains important when serious illness develops (Luks et al., 2024).

These principles remain the foundation of altitude-illness prevention. The unresolved problem is whether they are sufficient for contemporary rapid-ascent mountain sports, where ideal ascent profiles may be difficult to implement. When gradual ascent is impractical, some athletes and recreational participants may seek additional preparation before departure. Hypoxic pre-acclimatization has been studied for decades, including intermittent exposure and simulated-altitude protocols. The evidence is heterogeneous, however, and research protocols differ substantially in exposure dose and timing (Burtscher et al., 2022, Burtscher et al., 2026). The concept itself is established. What remains uncertain is how to develop a short protocol that fits contemporary rapid-ascent travel. A recent military study tested normobaric hypoxic inhalation before high-altitude deployment and reported a marked reduction in AMS incidence (Miao et al., 2026). Accordingly, this opinion uses that study as a timely example and argues for civilian research on practical pre-ascent preparation.

## 2 The prevention gap in rapid-ascent mountain sports

That agenda begins with a familiar prevention gap. The central principle of altitude-illness prevention is to avoid ascending too high too quickly. Current guidance recommends limiting sleeping-altitude gain above approximately 3,000 m and adding acclimatization days when possible. It also advises reduced exertion soon after arrival and consideration of acetazolamide when abrupt ascent cannot be avoided (Hackett and Shlim, 2025). These recommendations reflect the time required for acute acclimatization. During the first several days at altitude, ventilatory and cardiovascular responses adjust, oxygenation may improve, and tolerance of activity generally becomes better (Richalet et al., 2024).

Travel schedules do not always allow this progression. A person may fly to a plateau city and hike the next day, sleep at a high resort shortly after arrival, or drive rapidly to a high trailhead before meaningful acclimatization has occurred. This pattern is increasingly relevant to mountain sports because transport can shorten ascent time without shortening the biological process of acclimatization. Gradual ascent remains the safest approach, but it cannot always be incorporated into short trips or fixed event schedules.

Pharmacological prophylaxis can reduce AMS risk, but it does not remove the need for acclimatization or appropriate ascent behavior (Luks and Swenson, 2008; van Veelen et al., 2025). Some travelers also reach altitude without pre-travel medical consultation or prefer to avoid medication. Pre-ascent hypoxic preparation may therefore have a supplementary role if a practical protocol is shown to be effective and safe. The relevant research question is whether useful acclimatization can be induced before departure without encouraging participants to ignore established prevention after arrival.

## 3 A timely military signal for civilian research

Military altitude medicine offers useful evidence for this question because rapid deployment can create a problem similar to rapid-ascent travel. Personnel may move from low altitude to a high-altitude location and then face physical demands before acclimatization is complete. Military populations are more selected and more closely supervised than most civilian travelers, so their results cannot be transferred directly. Their experience can nevertheless help identify protocols that are practical enough to test in civilian settings.

Miao et al. (2026) studied aviation personnel preparing for high-altitude deployment. They used a normobaric hypoxic gas mixture containing approximately 14.2% oxygen, which was intended to simulate an altitude of about 3,400 m. Participants completed 1-h exposure sessions, with the full regimen lasting 7 days. The authors reported that oxygen saturation during hypoxic exposure improved across the training period, while the hypoxia-related increase in heart rate became smaller. Most importantly, they reported an AMS incidence of 28.5% in untrained personnel and 2.1% after 7 days of training, a relative reduction of 92.6%.

These findings do not establish a civilian protocol. The participants were selected military personnel, and the conditions of supervision differ from ordinary travel. The study is nevertheless timely because the regimen was brief and designed around an operational need for rapid ascent. It shows that a short pre-departure protocol can be implemented before real high-altitude exposure and then evaluated after arrival. For civilian research, the next question is whether a similarly practical approach produces reliable benefit under travel and mountain sports conditions.

## 4 What should hypoxic preparedness mean physiologically?

Pre-ascent hypoxic preparedness should be treated as a research concept rather than as a single physiological state. A change during hypoxic exposure does not by itself demonstrate protection after ascent. Improved SpO_2_ or a smaller heart-rate response may indicate adaptation to the training stimulus, but these measures are not substitutes for clinical assessment of AMS. They also cannot establish protection against high-altitude cerebral edema or high-altitude pulmonary edema.

Civilian trials should therefore assess efficacy after a defined ascent. The Lake Louise AMS Score provides a validated basis for measuring AMS symptoms (Roach et al., 2018). Physiological measures can then help explain why participants differ in their response. Resting and exertional SpO_2_, heart rate, blood pressure, and sleep oxygenation are relevant candidates. Functional testing is also important because mountain sports usually involve activity soon after arrival, when exercise tolerance may still be impaired (Cornwell et al., 2021; Richalet et al., 2024).

This distinction matters when interpreting a brief hypoxic protocol. A participant who shows better oxygenation during the final training session may still develop AMS after rapid ascent. A modest physiological response during training also does not prove that the protocol has failed. The primary test should be whether the intervention reduces clinically defined AMS after a standardized ascent. Physiological and functional outcomes can then be used to characterize the response without treating them as validated surrogate endpoints.

A useful civilian trial would therefore link the preparation period to the conditions that follow it. Symptom assessment should occur after ascent at prespecified times. Physiological measurements should be repeated after arrival, including during a standardized submaximal task when appropriate. This design would make it possible to examine whether changes observed during pre-acclimatization persist and whether they matter when participants begin to move at altitude.

## 5 From promising signal to civilian evidence

Defining the target state is only the first step; the next challenge is determining how much hypoxic exposure is required to produce it. The exposure dose remains uncertain. “Seven days” describes the schedule used by Miao et al. (2026), but it does not define a transferable dose. Simulated altitude, session duration, cumulative exposure, and the interval between sessions can all influence the response. A recent review of controlled simulated-altitude studies reported cumulative exposures from 7 to 104 h at simulated altitudes from 2,200 to 5,000 m. Reported reductions in AMS risk also varied widely from 12–73% (Burtscher et al., 2026). This range shows why the practical appeal of a 1-h/day protocol should be tested rather than assumed.

The persistence of any benefit is equally important. The military study does not establish a decay rate after the final hypoxic session, and a specific duration of protection should not be inferred from its results. Civilian studies should report the time between the final exposure and ascent as part of the intervention. Trials can also compare different intervals or repeat physiological assessment before departure and after arrival. This would show whether a short regimen remains relevant when travel itself delays exposure to altitude.

Field relevance also needs to be demonstrated. Normobaric hypoxia does not reproduce every feature of a mountain environment, and resting laboratory exposure may not predict what happens during early activity at altitude. Studies should therefore include a standardized exertional task after ascent and, when feasible, confirm laboratory findings during real mountain travel. Initial trials should focus on appropriately screened healthy adults who are likely to undertake rapid-ascent activities. Safety monitoring should include clear stopping criteria for excessive desaturation, concerning symptoms, or abnormal cardiovascular responses. Broader civilian populations should be studied only after basic efficacy and safety have been established.

The purpose of future research is to determine whether a brief and feasible protocol adds useful protection to current practice. It should be evaluated as an adjunct to guideline-based ascent planning rather than as permission to ascend more aggressively. **Table 1** summarizes the main questions that should be resolved before civilian use is considered.

**Table 1:** Research priorities for civilian evaluation of brief pre-ascent hypoxic preparation for mountain sports.

Research question	Mountain-sport relevance	Physiological or safety target	Recommended study design
What is the minimum effective exposure, and how long does the effect persist?	Travel schedules may allow only a few days of preparation, and ascent may occur hours or days after the last session.	AMS after ascent; SpO_2_ and heart-rate response; retention between the final exposure and ascent.	Compare sham, brief, and longer protocols. Prespecify the final-session-to-ascent interval and repeat assessments when feasible.
Does preparation improve early tolerance of activity after ascent?	Many travelers hike, ski, climb, or carry loads soon after arrival.	Exertional SpO_2_, perceived exertion, heart-rate response, and standardized walking or uphill performance.	Include a standardized submaximal task after ascent rather than relying on resting outcomes alone.
Can responses during hypoxic exposure identify people who do not tolerate or benefit from the protocol?	A short protocol is unlikely to produce the same response in every participant.	Excessive desaturation, symptoms during exposure, and atypical cardiovascular responses.	Relate within-person responses during preparation to post-ascent AMS and adverse events.
What safety limits are required before civilian use?	Civilian users are less selected than military personnel, and later studies may include people with relevant medical risk.	Clinically concerning symptoms, marked hypoxemia, and abnormal blood-pressure or heart-rate responses.	Begin with screened healthy adults and prespecify monitoring and stopping criteria before broadening eligibility.
Do normobaric protocols transfer to real mountain settings?	Laboratory hypoxia does not reproduce the complete environment or the activity demands of mountain travel.	AMS, sleep, oxygenation, exertional tolerance, and adverse events after real ascent.	Confirm promising laboratory protocols during standardized field ascents at defined sleeping altitudes.
How should hypoxic preparation complement established prevention?	Participants may overestimate protection and ascend more aggressively after completing a protocol.	Residual AMS risk and adherence to recommended ascent behavior.	Evaluate hypoxic preparation as an adjunct to gradual ascent and guideline-based prevention, not as a replacement.

## 6 Discussion

The need for renewed civilian research comes from the way people now reach altitude. Many can ascend quickly and begin activity soon after arrival, while practical pre-acclimatization protocols remain uncertain. Existing research provides the physiological basis for this work. The recent military study adds a timely field example because it tested a short regimen in a real rapid-ascent setting and reported a large difference in AMS incidence (Miao et al., 2026).

That observation is not sufficient for public recommendation. Civilian studies still need to establish whether brief normobaric exposure reduces clinically defined AMS after standardized rapid ascent and whether any benefit persists long enough to survive normal travel delays. The protocol also needs to be tested under early exertion, since that is when many travelers expect to be active. Until such evidence is available, gradual ascent and established medical prevention remain the basis of altitude-illness risk reduction (Luks et al., 2024). A brief pre-ascent protocol may eventually become an additional option, but its value should be demonstrated under the conditions in which civilian travelers would actually use it.
